# Top 10 Resources in Global Surgery

**DOI:** 10.9745/GHSP-D-20-00050

**Published:** 2020-09-30

**Authors:** Alliance Niyikuri, Emily R. Smith, Dominique Vervoort, Mark G. Shrime, Stav Brown, Alexander W. Peters, Gavin Yamey, Emmanuel Makasa

**Affiliations:** a Frank Ogden Medical School, Hope Africa University, Bujumbura, Burundi.; b Duke Global Health Institute, Duke University, Durham, NC, USA.; c Department of Public Health, Robbins College of Health and Human Sciences, Baylor University, Waco, TX, USA.; d Program in Global Surgery and Social Change, Harvard Medical School, Boston, MA, USA.; e Center for Global Surgery Evaluation, Massachusetts Eye and Ear Infirmary, Boston, MA, USA.; f Sackler School of Medicine, Tel Aviv University, Tel Aviv, Israel.; g Department of Surgery, Weill Cornell Medical College, New York, NY, USA.; h Cabinet Office, Government of the Republic of Zambia, Lusaka, Zambia.; i Department of Surgery, School of Medicine, University of Witwatersrand, Johannesburg, South Africa.

## Abstract

This resource list could serve to orient those interested in global surgery and could be supplemented with resources advocating for global surgery from clinical, population health, or policy perspectives.

## Key Messages

- The most useful resource reported was Global Surgery 2030, along with other publications, data collection tools, books and training manuals, and a documentary.
- This list could serve as a starting point for individuals interested in global surgery and be supplemented with resources advocating for global surgery from clinical, population health, or policy perspectives.

## THE GROWING FIELD OF GLOBAL SURGERY

Global surgery is defined as^1^:

*an area of study, research, practice, and advocacy that seeks to improve health outcomes and achieve health equity for all people who need surgical and anesthesia care, with a special emphasis on underserved populations and populations in crisis.*

The need is great. Surgical disease is among the top 15 causes of disability, and surgical conditions account for up to 30% of total disability-adjusted life years (DALYs) lost worldwide—with the greatest need in low- and middle-income countries (LMICs).^2^ Surgery has been shown to be highly cost-effective when compared with standard global health interventions.^3^^–^^5^

The transition from the Millennium Development Goals to the Sustainable Development Goals has usheredin a new era for the global surgery community. Sustain-able Development Goal 3, to “ensure healthy lives and promote well-being at all ages,” emphasizes health system strengthening and universal health coverage.^6^ The provision of available, accessible, safe, timely, and affordable surgical and anesthesia care is identified as an integral component of a functional health system in countries at all levels of economic development and as essential to achieving universal health coverage.^2^^,^^3^^,^^7^ In addition, the importance of increasing education, safety, and capacity for the provision of surgical, anesthetic, and obstetric care is highlighted by several global health and development agencies and policy makers, including the World Bank and the World Health Organization (WHO).^8^^–^^11^

As a result, the emerging field of global surgery has increased in priority among health practitioners, including nonphysician surgeons and anesthetists, researchers, and students. Evidence of this prioritization includes a shift toward incorporating surgical care as an integral part of global health systems strengthening in LMICs that has occurred and will likely continue to grow in importance within global health agendas.^12^^,^^13^ Lastly, interest in the field from an academic research standpoint is evidenced by the increase in peer-reviewed publications. Between 2005 and 2015, research publications in the field of global surgery increased from approximately 570 articles in 2005 to more than 4,000 articles published in 2015, according to PubMed.

Because of the growing interest in global surgery, momentum in this emerging field, and the importance of global surgery in the training of health professionals, we aimed to summarize the top resources in global surgery to orient readers to the field. We undertook a 2-stage process to identify and select the top 10 resources in global surgery.

In the first stage, we convened a team of global surgery leaders, including persons with decades of experience in global surgery and emerging leaders. The team included 6 global surgeons from a variety of surgical specialties and training and 2 public health professionals. A diverse authorship team was deliberately selected so that the nominated resources would transcend disciplines, career stages, and setting. The authorship team also included representation from varying career stages, including 2 students, 2 residents, 3 practicing surgeons, and 2 academic researchers. Lastly, the team included leaders from LMICs and high-income countries (HICs). Twenty-one resources were nominated de novo, including 10 peer-reviewed articles, 4 books or monographs, 3 data collection tools, 2 development manuals, and 2 advocacy pieces, comprising a documentary film and a newspaper opinion-editorial article.

The second stage involved a collaborative crowdsourcing initiative using social media and email distribution lists over a 1-month period. Using the social media and email platforms, we aimed to collect information from persons involved in global surgery from several perspectives, including surgeons, anesthesiologists and anesthetists, obstetricians, and public health students. We created a short survey that contained the 21 resources from stage 1 and asked participants to select their top 10 resources from this list. Participants were also asked to nominate other resources related to global surgery through short-answer responses. After the 1-month period, ratings and open-ended responses were compiled and tabulated, resulting in the nominated top 10 resources in global surgery.

## GLOBAL SURGERY TOP 10 RESOURCES

**FIGURE 1.**
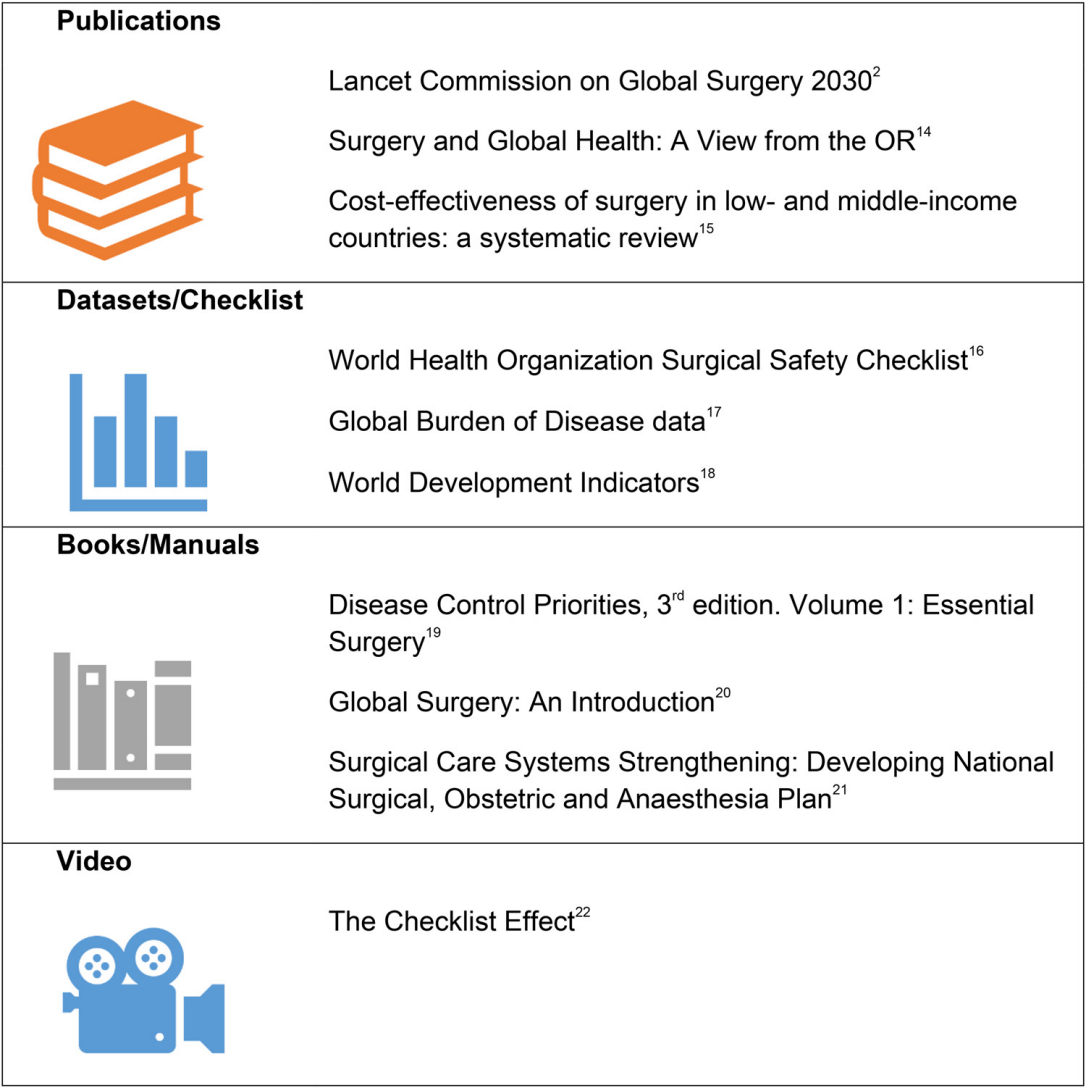
Top 10 Resources in Global Surgery

The top 10 resources included a wide variety of items including 3 publications, 3 data collection tools, 3 books or manuals, and 1 documentary film (Figure 1).

### Publications

Surgery and Global Health: A View from Beyond the OR (2008) is widely regarded as the impetus for global surgery to become a major global health emphasis.^14^ Written by 2 key leaders in global health, Dr. Paul Farmer and Dr. Jim Kim outlined the need for surgery in LMICs and defined global surgery as the “neglected stepchild of global health.”

Global Surgery 2030, a report by The *Lancet* Commission on Global Surgery (2015), repeatedly topped lists as the most useful and impactful resource.^2^ Launched in 2014, the Commission comprises a multidisciplinary, international team of commissioners, collaborators, and advisors from more than 110 countries and 6 continents. Its report, released in the following year, proposed a plan of action for this scale-up over the next 15 years. The data created by the Commission’s researchers have been included in the World Bank Development Indicators data collection and served as a launching point for the development of national surgical plans and the collection of primary global surgery data.

In 2014, Grimes and colleagues wrote Cost-Effectiveness of Surgery in Low- and Middle-Income Countries: A Systematic Review to evaluate the cost-effectiveness of surgical interventions that could be available at district hospitals in LMICs.^15^ The authors argued that many surgical interventions are as cost-effective, lifesaving, and disability preventing as traditional global public health measures (e.g., vaccination, bed nets for malaria prevention), thus debunking the myth that the provision of surgery is too expensive for low-resource settings.

### Datasets/Data Collection Tools

As part of the Safe Surgery Saves Lives initiative formed by the World Alliance for Patient Safety and WHO, the *WHO Surgical Safety Checklist* was created to assist surgical teams in reducing avoidable surgical site infections and preventable deaths.^16^ The checklist, which has been implemented in over 4,100 hospitals and still actively used in more than 1,790 hospitals, is associated with reduced complications, infection, and mortality rates, improved team communication, and increased economic benefits.^16^^,^^22^^,^^23^

The second data collection tool resource is the online Global Burden of Disease (GBD) data (2017) repository and visualization tools.^17^ The GBD Results Tool, hosted by the Institute for Health Metrics and Evaluation, allows for a comprehensive overview of disease morbidity and mortality around the world and comparison between countries, risk and causal factors, and patient demographics.

The final data collection tool resource includes the 6 core indicators reported by the *Lancet* Commission report that were incorporated in the World Bank Development Indicators in 2015.^18^ The incorporation of surgery-specific health indicators in the World Bank database, the largest collection of health, development, and economic data from members states, represents the first attempt to comprehensively gather primary surgical data on an international scale. In addition, the open-access nature of the data encourages accessibility to a wide range of users rather than only academics or those with sufficient economic resources.

### Books and Manuals

The third edition of the *Disease Control Priorities (DCP3)* series provides “the most up-to-date evidence on cost-effective interventions to address the burden of disease in low-resource settings.”^19^ Volume 1 of *DCP3* is dedicated to *Essential Surgery* and evaluates 44 emergency and essential surgical procedures.

*Global Surgery: An Introduction*, a booklet written by Dr. Dominique Vervoort in 2017, provides an introduction on the basic concepts, history, and future steps of global surgery and related topics, targeting an audience new to the field of global surgery.^20^

Lastly, following the movement toward National Surgical, Obstetric, and Anesthesia Plans (NSOAPs) in some sub-Saharan African countries and the launch of Zambia’s NSOAP, the WHO produced the *Surgical Care Systems Strengthening: Developing National Surgical, Obstetric, and Anaesthesia Plans* to highlight the importance of NSOAPs and the progress made in selected countries around the world.^21^

### Documentary

Finally, The Checklist Effect, a 2015 documentary by the Lifebox Foundation directed by Lauren Anders Brown, follows surgical health professionals in Haiti, Uganda, Mongolia, Guatemala, and Moldova.^22^ The documentary has been screened and awarded at several international global health film festivals and translated into 10 languages by student members of the International Student Surgical Network, an international nonprofit organization, consisting of medical students, residents, and young doctors interested in global surgery.

## ADDITIONAL RESOURCES

Due to the increased interest in global surgery among academic institutions and LMIC health care professionals, there is not only the need for a collection of first-step resources in the field but also the need for thoughtful guidelines to improve surgical care in LMICs and low-resource settings through multidisciplinary approaches.

**FIGURE 2.**
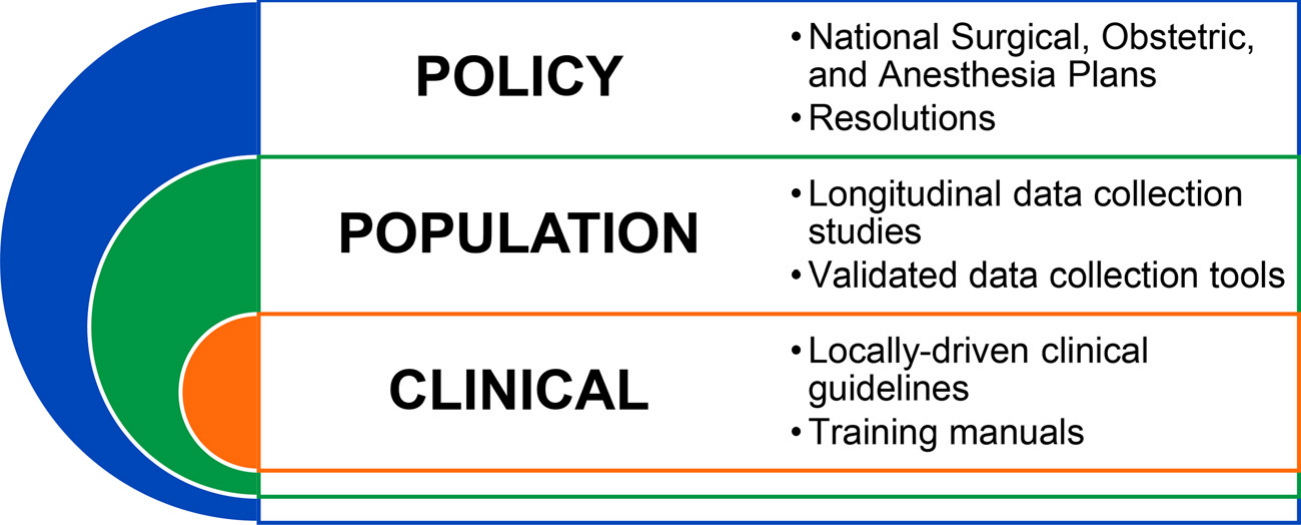
Socioecological Model of Additional Global Surgery Resource Recommendations

There are many additional resources, including publications, partnerships, training tools, and ongoing studies on global surgery that are moving the field forward in tremendous ways. This list is not meant to be comprehensive or final, but rather a starting point for students, professionals, clinicians, and others interested in global surgery. Initially, the focus of interest in global surgery has solely been on providing clinical care to persons with surgical conditions. However, the field has broadened to include public health, health economics, health services, and policy research perspectives, thus promoting the need for global surgery resources to encompass both the individual-level and population-level needs, while contextually incorporating locally-driven viewpoints. Building upon the top 10 resources, other key supplementary resources from a socioecological viewpoint are described below (Figure 2).

### Clinical Perspective

On a clinical level, the field of global surgery is seeing momentum build around clinical guidelines for care in resource-constrained settings that are relevant to practitioners in LMICs. Some clinical practice resource examples include *Primary Sur-gery*, *Principles of Reconstructive Surgery in Africa*, *Paediatric Surgery: A Comprehensive Textbook for Africa*, SUGAR PEARLS (Procedural Education for Adaptation to Resource Limited Settings*)*, and many more.^24^^–^^27^ In addition, locally driven surgical skills and training resources, such as *Global Surgery and Anesthesia Manual* and *Non-Technical Skills for Surgeons System Handbook* have also been made available to health practitioners in LMICs.^28^^,^^29^

Additional effects toward trauma care improvement, including prehospital and emergency room care, have also been emphasized from a full health care system perspective.^30^ Although prehospital chains (e.g., through ambulance services or clear referral systems) and emergency services remain scant and under-addressed in global health, they form critical barriers in access to care by increasing the delay in reaching care.^31^ For example, emergency medical services systems are in place in only one-third of the countries in sub-Saharan Africa, only covering less than 10% of the population.^32^ Lastly, but importantly, nonphysician clinicians and nonspecialist physicians can also assist in closing the gap in needed surgical services at first-level hospitals.^33^ A recent review by Falk et al. emphasized the variation in recognition, training pathways, and procedural support that nonspecialist and nonclinician providers engage in through task-sharing models.^34^ Task sharing has accordingly become a predominant approach in surgical care delivery in many LMICs with formalized training pathways in, for example, Mozambique and Sierra Leone.

As a recommendation for the development of future resources, international collaborative efforts and partnerships (whether researchers in both HICs and LMICs or researchers in LMICs among themselves) can guide the creation of global surgery resources that cater to the needs of local stakeholders among practitioners in LMICs. Trainees and surgeons in LMICs must be fully involved in these endeavors and their need for more specific and targeted clinical resources addressed as well. Though already exposed to care in resource-constrained settings, trainees in LMICs need clinical guidelines such as those previously cited as they prepare to care for patients in their local low-resource settings. Reciprocity with collaborative partners must be ensured, sharing clinical experience from HICs and learning new approaches to surgical disease already practiced by local surgeons.^35^

### Population Health Perspective

On a population health level, collaborative efforts resulting in the collection of primary epidemiologic data are needed. Several large, multinational cohort studies on surgery, such as GlobalSurg, Global PaedSurg, COVIDSurg and other national and international databases on trauma care, neurosurgery, pediatric surgery, and other specialty areas, continue to collect primary data in LMICs, which has resulted in several profound publications highlighting the global surgery need from an epidemiologic standpoint.

In addition to the longitudinal cohort studies, there are several data collection tools available for population-level data collection, including the aforementioned tools used in the Global Burden of Disease tools and the World Bank Development Indicators. Other data collection tools include the Surgeons Overseas Assessment of Surgical Need, the Surgeons Overseas PIPES—Personnel, Infra-structure, Procedure, Equipment, and Supplies—Assessment tool, Surgeons Overseas Pediatric PIPES, the Global Assessment of Pediatric Surgery, and the World Health Organization Tool for Situational Analysis to Assess Emergency and Essential Surgical Care.

Recommendations for future resources from a population health perspective include additional longitudinal studies that are contextually applicable to specific populations. Validated questionnaires assessing a more granular approach to measure the burden of surgical conditions, facility-level tools to measure quality of care, and qualitative data collection tools aimed at overcoming barriers to surgical care are a few examples of further tools to be developed.

### Policy Perspective

Strong support for strengthening emergency and essential surgical and anesthesia care has been advocated as an essential component of universal health coverage and was recognized by the World Health Assembly (WHA) as resolution WHA68:15. In 2017, another resolution was passed (WHA70.22) that requires member nations to provide biennial reports on the strengthening of emergency and essential surgical care and anesthesia as a component of universal health coverage as requested in resolution WHA69:11. This series of global events at the policy level of health care highlight the role that policy makers and advocates of global surgery have on the international health agenda.

The promotion of recommendations to develop national surgical plans have propelled many countries to engage local stakeholders at various levels of health care and policy to begin formulating NSOAPs. Many organizations and institutions have been involved in engaging key stakeholders to advocate for the surgical patient. As an example, the G4 Alliance, comprising over 80 organizations in more than 160 countries, has played a strong role in advocacy, policy implementation, and strategic planning for global surgery as outlined in their forthcoming Global Action Plan for Surgery.^36^ Another example includes the Optimal Resources for Children’s Surgery, developed by the Global Initiative for Children’s Surgery, a collaboration of more than 270 pediatric health care professionals and academicians from 44 countries (75% from LMICs).^37^^,^^38^ The document outlined detailed standards of children’s surgical care at all levels of health care facilities, with the objective to inform national surgical plans in LMICs.

## CONCLUSION

We identified key resources as relevant to practitioners from various specialties and stages of their careers and listed the top 10 resources relevant to individuals across all groups. We hope that this list will serve as a starting point for individuals interested in global surgery and aid in promoting global surgery awareness, scholarship, and collaboration toward a world in which safe surgical and anesthesia care is a reality for all.

## Notes

**Peer Reviewed**

## References

[1] Byass P. The imperfect world of global health estimates. PLoS Med. 2010;7(11):e1001006. 10.1371/journal.pmed.1001006. 21152416 PMC2994666

[2] Meara JG, Leather AJM, Hagander L, et al. Global Surgery 2030: evidence and solutions for achieving health, welfare, and economic development. Lancet. 2015;386(9993):569–624. 10.1016/S0140-6736(15)60160-X. 25924834

[3] Smith ER, Concepcion TL, Niemeier KJ, Ademuyiwa AO. Is global pediatric surgery a good investment? World J Surg. 2019;43(6):1450–1455. 10.1007/s00268-018-4867-4. 30506288

[4] Saxton AT, Poenaru D, Ozgediz D, et al. Economic analysis of children’s surgical care in low- and middle-income countries: a systematic review and analysis. PLoS One. 2016;11(10):e0165480. 10.1371/journal.pone.0165480. 27792792 PMC5085034

[5] Chao TE, Sharma K, Mandigo M, et al. Cost-effectiveness of surgery and its policy implications for global health: a systematic review and analysis. Lancet Glob Health. 2014;2(6):e334–e345. 10.1016/S2214-109X(14)70213-X. 25103302

[6] United Nations. Sustainable Development Goals. Accessed August 11, 2020. https://www.un.org/sustainabledevelopment/sustainable-development-goals/

[7] Mathers CD, Loncar D. Projections of global mortality and burden of disease from 2002 to 2030. PLoS Med. 2006;3(11):e442. 10.1371/journal.pmed.0030442. 17132052 PMC1664601

[8] Abdullah F, Troedsson H, Cherian M. The World Health Organi-zation program for emergency surgical, obstetric, and anesthetic care: from Mongolia to the future. Arch Surg. 2011;146(5):620–623. 10.1001/archsurg.2011.84. 21576615

[9] Bickler SW, Spiegel DA. Global surgery—defining a research agenda. Lancet. 2008;372(9633):90–92. 10.1016/S0140-6736(08)60924-1. 18582930

[10] Hedges JP, Mock CN, Cherian MN. The political economy of emergency and essential surgery in global health. World J Surg. 2010;34(9):2003–2006. 10.1007/s00268-010-0610-5. 20454792

[11] Peters AW, Pyda J, Menon G, Suzuki E, Meara JG. The World Bank Group: innovative financing for health and opportunities for global surgery. Surgery. 2019;165(2):263–272. 10.1016/j.surg.2018.07.040. 30274731

[12] Citron I, Meara JG. The evolving role of surgery in the international public health agenda. Cad Saude Publica. 2017;33(10):e00124217. 10.1590/0102-311x00124217. 29091174

[13] Spiegel DA, Misra M, Bendix P, et al. Surgical care and health systems. World J Surg. 2015;39(9):2132–2139. 10.1007/s00268-014-2928-x. 25561195

[14] Farmer PE, Kim JY. Surgery and global health: a view from beyond the OR. World J Surg. 2008;32(4):533–536. 10.1007/s00268-008-9525-9. 18311574 PMC2267857

[15] Grimes CE, Henry JA, Maraka J, Mkandawire NC, Cotton M. Cost-effectiveness of surgery in low- and middle-income countries: a systematic review. World J Surg. 2014;38(1):252–263. 10.1007/s00268-013-2243-y. 24101020

[16] World Health Organization (WHO). *Surgical Safety Checklist*. WHO; 2009. Accessed August 11, 2020. https://apps.who.int/iris/bitstream/handle/10665/44186/9789241598590_eng_Checklist.pdf

[17] Institute for Health Metrics and Evaluation. Global Burden of Disease data. Accessed August 11, 2020. http://www.healthdata.org/gbd/data

[18] World Bank. World Development Indicators. World Bank; 2010. Updated July 1, 2020. Accessed August 11, 2020. https://datacatalog.worldbank.org/dataset/world-development-indicators

[19] Debas HT, Donkor P, Gawande AA, Jamison DT, Kruk ME, Mock CN, eds. *Disease Control Priorities, Third Edition (Volume 1): Essential Surgery*. The International Bank for Reconstruction and Development, The World Bank; 2015. 10.1596/978-1-4648-0346-8. 26740991

[20] Vervoort D. *Global Surgery: An Introduction*. InciSioN; 2017. Accessed August 11, 2020. https://issuu.com/dominiquevervoort/docs/global_surgery-_an_introduction

[21] World Health Organization (WHO). *Surgical Care Systems Strengthening: Developing National Surgical, Obstetric and Anaesthesia Plans*. WHO; 2017. Accessed August 11, 2020. https://apps.who.int/iris/bitstream/handle/10665/255566/9789241512244-eng.pdf

[22] Brown L. The Checklist Effect. 2015.

[23] Tscholl DW, Weiss M, Kolbe M, et al. An anesthesia preinduction checklist to improve information exchange, knowledge of critical information, perception of safety, and possibly perception of teamwork in anesthesia teams. Anesth Analg. 2015;121(4):948–956. 10.1213/ANE.0000000000000671. 25806399

[24] King M, Awori N, Bewes P, et al, eds. *Primary Surgery Volume Two: Trauma*. Oxford University Press; 1987.

[25] Carter LL Jr, ed. *Principles of Reconstructive Surgery in Africa*. Pan-African Academy of Christian Surgeons; 2013.

[26] Ameh E, Bickler S, Lakhoo K, et al, eds. *Paediatric Surgery: A Comprehensive Textbook for Africa*. Global HELP; 2011. https://global-help.org/products/paediatric_surgery_a_comprehensive_text_for_africa/

[27] Board of Regents of the University of Wisconsin System. SUGAR PEARLS. Accessed August 11, 2020. https://sugarprep.org/pearls/

[28] Meara JG, McClain CD, Mooney DP, eds. *Global Surgery and Anesthesia Manual: Providing Care in Resource-Limited Settings*. Taylor & Francis Group; 2014.

[29] The University of Aberdeen. *The Non-Technical Skills for Surgeons (NOTSS) System Handbook v1.2*. University of Aberdeen; 2012. Accessed August 11, 2020. https://www.rcsed.ac.uk/media/4605/notss-handbook-2012-no-bleeds.pdf

[30] Mock CN, Jurkovich GJ, nii-Amon-Kotei D, Arreola-Risa C, Maier RV. Trauma mortality patterns in three nations at different economic levels: implications for global trauma system development. J Trauma. 1998;44(5):804–812. 10.1097/00005373-199805000-00011. 9603081

[31] Ouma PO, Maina J, Thuranira PN, et al. Access to emergency hospital care provided by the public sector in sub-Saharan Africa in 2015: a geocoded inventory and spatial analysis. Lancet Glob Health. 2018;6(3):e342–e350. 10.1016/S2214-109X(17)30488-6. 29396220 PMC5809715

[32] Mould-Millman NK, Dixon JM, Sefa N, et al. The state of emergency medical services (EMS) systems in Africa. Prehosp Disaster Med. 2017;32(3):273–283. 10.1017/S1049023X17000061. 28228178

[33] Ashengo T, Skeels A, Hurwitz EJH, Thuo E, Sanghvi H. Bridging the human resource gap in surgical and anesthesia care in low-resource countries: a review of the task sharing literature. Hum Resour Health. 2017;15(1):77. 10.1186/s12960-017-0248-6. 29115962 PMC5688799

[34] Falk R, Taylor R, Kornelsen J, Virk R. Surgical task-sharing to non-specialist physicians in low-resource settings globally: a systematic review of the literature. World J Surg. 2020;44(5):1368–1386. 10.1007/s00268-019-05363-7. 31915975

[35] Ozgediz D, Wang J, Jayaraman S, et al. Surgical training and global health: initial results of a 5-year partnership with a surgical training program in a low-income country. Arch Surg. 2008;143(9):860–865. 10.1001/archsurg.143.9.860. 18794423

[36] The G4 Alliance. G4 Alliance. Accessed August 19, 2020. http://www.theg4alliance.org

[37] Global Initiative for Children’s Surgery. Global Initiative for Children’s Surgery: A Model of Global Collaboration to Advance the Surgical Care of Children. World J Surg. 2019;43(6):1416–1425. 10.1007/s00268-018-04887-8. 30623232 PMC7019676

[38] Global Initiative for Children’s Surgery. Optimal resources for child-ren’s surgical care: executive summary. World J Surg. 2019;43(4):978–980. 10.1007/s00268-018-04888-7. 30725368

